# Long-term Outcome of Unruptured Cerebral Aneurysms

**DOI:** 10.2188/jea.13.289

**Published:** 2007-11-30

**Authors:** Eiji Matsumoto, Toshio Masuzawa, Yosikazu Nakamura

**Affiliations:** 1 Department of Surgical Neurology, Jichi Medical School.; 2 Department of Public Health, Jichi Medical School.; 3 Dr. Masuzawa died September 8, 2003.

**Keywords:** aneurysms, unruptured, cerebral arterial diseases, cerebrovascular accident, observation, subarachnoid hemorrhage

## Abstract

BACKGROUND: The frequency at which unruptured cerebral aneurysms are detected has increased due to advances in low- or non-invasive diagnostic techniques. Despite the recent improvements in surgical and medical management of aneurysmal subarachnoid hemorrhages, however, the overall case-fatality rate of this disease is still high. To reduce it, the natural history of unruptured cerebral aneurysms should be better understood.

METHODS: The subjects consisted of 156 patients with unruptured cerebral aneurysms who had been admitted to the Department of Surgical Neurology, Jichi Medical School Hospital or Jichi Medical School Ohmiya Medical Center, Japan, between January 1989 and December 1998. All of the patients were classified according to the process by which aneurysms had been detected. The expected number of deaths was calculated by using age- and sex-specific mortality rates obtained from the vital statistics. A standardized mortality ratio and 95% confidence interval were calculated by using the expected and observed numbers of deaths.

RESULTS: The standardized mortality ratios differed among the groups according to the process of detecting aneurysms. The incidence rate of rupture of unruptured cerebral aneurysms was 1.3 per 100 person-years. The annual rupture rate of unruptured cerebral aneurysms was higher than previously reported. The survival rate of the group that underwent surgical treatment for unruptured cerebral aneurysms was higher than that of the untreated group.

CONCLUSIONS: The annual rupture rate of unruptured cerebral aneurysms may be higher than previously reported.

The frequency of detecting unruptured cerebral aneurysms (UCAs) has increased due to advances in low- or non-invasive diagnostic techniques, such as high resolution computed tomography (CT), magnetic resonance imaging (MRI) including magnetic resonance angiography (MRA), and digital subtraction angiography (DSA).^1^ In particular, a screening system for asymptomatic brain disease in healthy individuals, with the purpose of preventing cerebrovascular disease [including a subarachnoid hemorrhage (SAH)] is widely used throughout Japan; and UCAs are being identified with increasing frequency.^2^ Despite the recent improvements in surgical and medical management of aneurysmal SAH, the overall case-fatality rate for this disease is still high (about 40 to 50%).^3^ The consensus is that treatment of unruptured aneurysms depends on the relative risk of a subsequent aneurysmal rupture in untreated patients in relation to the risk of surgical treatment.

The natural history of UCAs has been controversial because of the selection bias of observed populations.^4^^-^^11^ The history of UCAs discovered incidentally in examinations of nonspecific symptoms or in screening should have less selection bias. The natural history of these UCAs is not well understood, however, because they have only recently become widely detected with the increasing use of MRA.

The present study was attempted to clarify statistically the clinical course of UCAs.

## METHODS

The subjects were selected from the 156 consecutive patients (56 males, 100 females; average age, 56.3 years; range: 28-79 years) with UCAs who had been admitted to the Departments of Surgical Neurology, Jichi Medical School Hospital or Jichi Medical School Ohmiya Medical Center, Japan, between January 1989 and December 1998. All of the patients underwent angiography and those with the following conditions were excluded from the present observation: fusiform or dissecting aneurysms; intra-cavemous lesions or small aneurysms in the paraclinoid area that were not well-distinguished from intracavernous aneurysms; small lesions that were indistinguishable from carotid infundibular dilatation or atherosclerotic changes; and the presence of a high risk in treating UCAs, such as severe systemic disorders or major neurological deficits. There is no difference in the frequency of systemic disorders and neurological symptoms between the treated and non-treated groups.

A surgical indication for aneurysms that were identified incidentally was deliberately determined by considering multiple factors in individual patients. The basic selection criteria for surgery were as follows: low surgical risk in terms of size and location of aneurysm, age under 70 years, favorable medical conditions, and no major neurological deficits due to associated diseases.

The aneurysms were grouped according to the process by which they were discovered: Group 1 consisted of those identified at the time of investigation of SAH due to the rupture of coexistent aneurysms; Group 2 was composed of aneurysms discovered incidentally during an intracranial investigation for systemic lesions other than SAH; Group 3 comprised of aneurysms that produced mass signs or ischemic symptoms; Group 4 consisted of purely incidental aneurysms that were first discovered at a routine examination with CT or MRI (including MRA) for nonspecific symptoms such as headache, dizziness, tinnitus, or numbness of limbs; and Group 5 was composed of purely incidental aneurysms discovered at a screening test, using non-invasive diagnostic testing such as MRA during a medical examination of the brain in seemingly healthy individuals. Patients with Group 1 aneurysms who had been treated with a single-stage surgical operation directed at all the aneurysms were excluded from this study.

All patients were observed from the time when the aneurysm was discovered until either death or December 1998. Medical records of the two institutions were reviewed, and the rupture of UCAs and deaths were checked. Patients who did not visit the institutions after January 1999 were asked about their status by mail or telephone. The expected number of deaths for this patient group was calculated by using age- and sex-specific mortality rates obtained from the vital statistics collated in 1992 for the period from 1989 through 1994, and those in 1997 for the period from 1995 through 1998. The standardized mortality ratios (SMRs) and their 95% confidence intervals (CIs) were calculated by using the expected and observed numbers of deaths. The deceased subjects were divided into two groups according to whether or not their aneurysms had ruptured; and the SMR was then calculated for each group. The age- and sex-specific mortality and morbidity rates from SAH due to the number of aneurysm ruptures per 100 person-years were calculated by using the observed person-years and the observed number of deaths. Data were analyzed statistically using the Kaplan-Meier methods and Mantel-Cox tests.

## RESULTS

The clinical backgrounds of the 156 eligible patients are summarized in Table 1. Of these, 108 underwent surgical treatment (treated group) and 48 underwent conservative treatment (non-treated group). Group 1 consisted of 41 cases; Group 2, 31; Group 3, 21; Group 4, 42; and Group 5, 21.

**Table 1.** Summary of 156 patients among those with unruptured cerebral aneurysms in Jichi Medical School, 1989-1998.

		total number(%)	treated group(%)	non-treated group(%)
Number of patients		156(100)	108(100)	48(100)
sex				
	male	56(36)	34(31)	22(46)
	female	100(64)	74(69)	26(54)
age(year)				
	-39	10(6)	7(7)	3(6)
	40-49	36(23)	24(22)	12(25)
	50-59	36(23)	25(23)	11(23)
	60-69	62(40)	46(43)	16(33)
	70-79	12(8)	6(6)	6(13)

How to detect the aneurysms				
	UCAs associated with subarachnoid hemorrhage (Group 1)	41(26)	27(25)	14(29)

	UCAs discovered incidentally during systemic investigations (Group2)	31(20)	23(21)	8(17)

	UCAs producing mass effects or thromboembolism (Group3)	21(14)	15(14)	6(13)

	UCAs discovered incidentally during investigations for symptoms unrelated to the UCAs (Group4)	42(27)	29(27)	13(27)

	UCAs discovered incidentally during the medical check of the brain (Group5)	21(14)	14(13)	7(15)

All 156 patients had been followed-up until the end of 1998 or their deaths. The observed number of deaths during the follow-up period was 22 (9 males and 13 females). The mortality rates of all groups are summarized in Table 2. Because all of the 7 patients who suffered from SAH died prior to treatment, the mortality and morbidity rates from aneurysmal rupture were equal. A list of the fatal cases is shown in Table 3. The SMR was 4.64 with a 95% CI of 2.91-7.01 (Table 4), the SMRs being significantly different among the 5 groups. All patients dying of SAH had absolute proof of the disease confirmed by CT. They were transferred to neurosurgical services but died from a massive SAH before treatment could be given.

**Table 2.** Summary of the mortality rate among those with unruptured cerebral aneurysms in Jichi Medical School, 1989-1998.

whole	observed person-year	number of deaths	mortality rate (/100 person-years)
	528.6	22	4.2
male			
female	205.5	9	4.4
	323.1	13	4.0
age (year)			
-59			
60-	265.3	5	1.9
	263.3	17	6.5
death from subarachnoid hemorrhage			
death from other than subarachnoid hemorrhage	528.6	7	1.3
	528.6	15	2.8

**Table 3.** A list of 22 fatal cases among those with unruptured cerebral aneurysms in Jichi Medical School, 1989-1998.

No.	age at aneurysm detection (year)	Sex	detection of aneurysm (Group)	age at death (years)	cause of death
1	45	M	1	50	SAH
2	46	M	2	47	Suicide
3	47	M	1	52	Suicide
4	47	F	2	49	Unknown
5	49	M	2	50	Intracerebral hemorrhage
6	61	F	1	61	Acute myocardiac infarction
7	62	F	2	63	Pneumonia
8	62	F	2	63	Unknown
9	63	M	2	65	Chronic renal failure
10	63	M	1	69	Ileus
11	64	F	1	66	Pneumonia
12	66	M	1	69	SAH
13	66	F	4	66	Asthma attack
14	67	F	2	68	Unknown
15	68	M	4	75	SAH
16	70	F	4	74	SAH
17	71	F	1	72	SAH
18	71	F	2	74	Heart failure
19	72	M	4	75	cerebral infarction
20	73	F	1	76	SAH
21	74	F	1	75	SAH
22	79	F	1	80	Pneumonia

According to the age at the aneurysm detection

SAH: subarachnoid hemorrhage

Group1: Unruptured cerebral aneurysms (UCAs) in multiple aneurysms in patients with subarachnoid hemorrhage (SAH).

Group2: UCAs discovered incidentally during investigation for systemic lesions other than SAH.

Group3: UCAs producing mass effects or thromboembolism.

Group4: UCAs discovered incidentally during investigations for symptoms unrelated to the UCAs.

Group5: UCAs discovered at a screening test using low- or non-invasive diagnostic study such as MRA in healthy individuals during the medical check up of the brain.

**Table 4.** The observed and expected numbers of death among those with unruptured cerebral aneurysms in Jichi Medical School, 1989-1998.

	n	number of death		standardized mortality ratio (95% confidence interval)

		observed	expected	
All subjects	156	22	4.74	4.64(2.91-7.01)

male	56	9	2.36	3.82(1.75-7.25)
female	100	13	2.38	5.47(2.91-9.35)

treated group				
male	34	3	1.44	2.08(0.43-6.07)
female	74	6	1.82	3.29(1.21-7.17)

non-treated group				
male	22	6	0.92	6.55(2.41-14.29)
female	26	7	0.55	12.64(5.07-26.04)

How to detect the aneurysms				
Group 1	41	10	1.85	5.39(2.59-9.92)
Group 2	31	8	1	8.04(3.46-15.83)
Group 3	21	0	0.65	0
Group 4	42	4	1.47	2.73(0.74-6.98)
Group 5	21	0	0.29	0

Group1: Unruptured cerebral aneurysms (UCAs) in multiple aneurysms in patients with subarachnoid hemorrhage (SAH).

Group2: UCAs discovered incidentally during investigation for systemic lesions other than SAH.

Group3: UCAs producing mass effects or thromboembolism.

Group4: UCAs discovered incidentally during investigations for symptoms unrelated to the UCAs.

Group5: UCAs discovered at a screening test using low- or non-invasive diagnostic study such as MRA in healthy individuals during the medical check up of the brain.

The mean age of the 108 patients in the treated group (34 males and 74 females) was 56.1 years (range, 30 to 76 years). Postoperative mortality was zero but the operative neurological complication was 5.6% (6 cases). Two patients suffered from permanent hemiparesis, 3 from a consciousness disturbance, and 1 presented with a hemorrhagic infarction. The follow-up period for these 108 patients ranged from 34 to 3,562 days (9.7 years), with an average of 1,236 days (3.4 years). The observed number of deaths was 9 (3 males and 6 females). The SMR for males was 2.08 (95% CI: 0.43-6.07), for females, 3.29 (95% CI: 1.21-7.17) (Table 4).

The mean age of the 48 patients in the non-treated group (22 males and 26 females) was 56.6 years (range, 28 to 79 years). The follow-up period for them ranged from 19 to 3,646 days (10 years), with an average length of 1,221 days (3.3 years). The observed number of deaths was 13 (6 males and 7 females). The SMR for males was 6.55 (95% CI: 2.41-14.3), and for females it was 12.64 (95% CI: 5.07-26.0) (Table 4). The causes of death are shown in Table 3. Seven patients died of SAH (395, 464, 1,069 (2.9 years), 1,329 (3.6 years), 1,597 (4.4 years), 1,959 (5.4 years), and 2,517 days (6.9 years) later). Of the 48 non-treated patients, 7 (15%) suffered from SAH due to an aneurysmal rupture. The morbidity of rupture, equal to the mortality rate as noted above, of SAH due to aneurysmal rupture was 4.4 per 100 person-years (Table 5).

**Table 5.** rupture rate of unruptured cerebral aneurysms in Jichi Medical School, 1989-1998.

		observed person-year	number of deaths	rupture rate (100 person-year)
total		528.6	7	1.3

male		205.5	3	1.5
female		323.1	4	1.2

age (years)				
-59		265.3	1	0.4
60+		263.3	6	2.3

treated group		368.5	0	0.0
non-treated group		160.1	7	4.4
	Group 1*	51.6	5	9.7
	Group 4 and Group 5*	58.9	2	3.4

* : secondary mention of non-treated group

Group1: Unruptured cerebral aneurysms (UCAs) in multiple aneurysms in patients with subarachnoid hemorrhage (SAH).

Group4: UCAs discovered incidentally during investigations for symptoms unrelated to the UCAs.

Group5: UCAs discovered at a screening test using low- or non-invasive diagnostic study such as MRA in healthy individuals during the medical check up of the brain.

**Figure 1.**
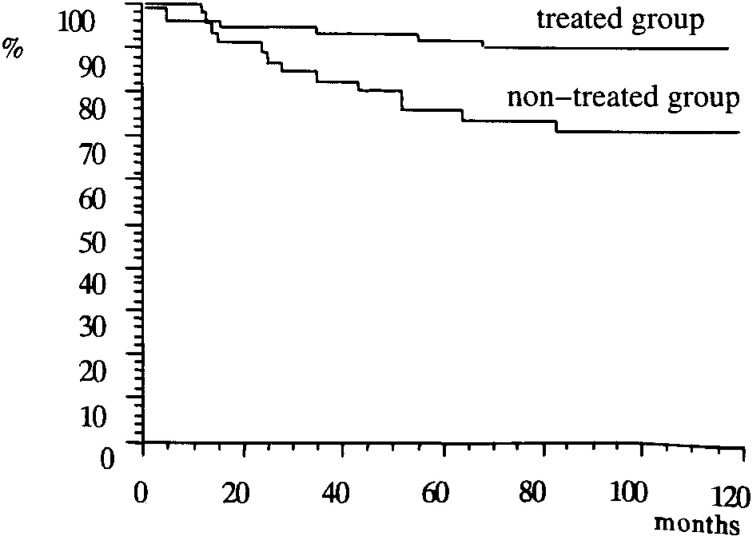
Survival curves of treated and non-treated group by Kaplan-Meier method. (p<0.01)

The survival curves for both the treated and non-treated groups are shown in Figure 1. The survival rate of the former was greater than for the latter. Treating the UCAs may improve the patients’ prognoses significantly.

As shown in Table 5, of the 7 patients who suffered from SAH due to an aneurysmal rupture, 5 belonged to the cohort of the 14 non-treated patients with aneurysmal Group 1; and their incidence rates from SAH due to aneurysmal rupture corresponded to 9.7 per 100 person-years. The other 2 patients had aneurysms that belonged to Group 4. Groups 4 and 5 had the least selection bias of the five groups in this study. Of the 20 non-treated patients in Groups 4 and 5, two suffered from SAH. Their morbidity rates from SAH due to aneurysmal ruptures corresponded to 3.4 per 100 person-years.

## DISCUSSION

The ideal therapy for UCAs with minimum surgical complications is surgery conducted before aneurysmal rupture. Appropriate treatment for UCAs may reduce the incidence of SAH. However, the natural course of UCAs has not been elucidated,^4^-^11^ and whether prophylactic surgery applied to UCAs reduces the incidence of SAH remains controversial. Before MRI and MRA had been developed, many UCAs were discovered by chance during angiography for SAH due to a rupture of another aneurysm or ischemic cerebral disease. The rupture rate of those aneurysms within one year was as high as 9.1-26%.^5^^,^^6^^,^^9^ In general, the annual rupture rate of UCAs is now believed to be 1- 1.7%.^8^^,^^13^^-^^15^ However, in 1998, The International Study of Unruptured Intracranial Aneurysms Investigators^12^ reported that in patients without a history of SAH, the annual rate of rupture was 0.05% for aneurysms less than 10 mm in diameter. Many questions have been raised concerning this study and the actual annual rate of rupture is still controversial.^16^

In the present study, the SMR of the treated group was lower than that of the non- treated group (Table 4), and the survival rate of the former was statistically higher than the latter (Figure 1). However, the SMR of the treated group was two or three times higher than the expected rate obtained from the vital statistics in Japan. This was accounted for by the high SMR of Group 2, which consisted of incidental aneurysms discovered during intracranial investigations for systemic lesions other than SAH, and the observed number of deaths was higher than the expected one.

The person-year method used to calculate the incidence rate of SAH due to aneurysmal rupture (Table 5) yielded 4.4 per 100 person-years in the non-treated group. However, this value contains a bias in that it contains UCAs that have a high risk of rupture, consisting of aneurysms identified at the time of investigation of SAH due to the rupture of a coexistent aneurysm (Group 1) and aneurysms producing mass signs or ischemic symptoms (Group 3). On the other hand, UCAs that were first discovered by routine CT or MRI (including MRA), performed for nonspecific symptoms such as headaches, dizziness, tinnitus, or limb numbness (Group 4), and those discovered at a screening test using non-invasive diagnostic testing such as MRA in seemingly healthy individuals during medical checkups of the brain (Group 5) have less biases. In Groups 4 and 5, 2 cases (10%) of the 20 non-treated UCA cases suffered from aneurysmal ruptures and the incidence rate of SAH due to aneurysmal rupture was 3.4 per 100 person-years (Table 5).

The cumulative incidence was calculated by using the natural log as follows:^17^

CI=1-e^−It^,

where CI denotes cumulative incidence, I, incidence rate, and t, the time of observation. Thus, when the incidence rate of aneurysmal rupture per 100 person-years over a 10-year observation period is 3.4, the cumulative incidence of aneurysmal rupture within 10 years is 0.288. This means that almost 29 of 100 subjects will suffer from an aneurysmal rupture during a 10-year observation period. Thus the annual rupture rate of UCAs may be higher than the figures that have been reported so far.

We were unable to evaluate whether surgical intervention against UCAs is effective in reducing the incidence of SAH because this was an observational study. A well-designed randomized controlled trial is required to address this issue. However, the present study did determine the incidence rates of aneurysmal ruptures and present survival curves for both the treated and non-treated groups; and it was found that aneurysmal ruptures were not observed in the treated group with none lost during surgery.

In conclusion, the incidence rate of SAH and the mortality rate among those with unruptured cerebral aneurysms were determined. These data suggested that the annual rupture rate of unruptured cerebral aneurysms may be higher than previously reported.
